# Deontological Guilt Differentially Affects Moral Behaviour in Participants With and Without Obsessive–Compulsive Disorder (OCD)

**DOI:** 10.1002/cpp.70252

**Published:** 2026-03-27

**Authors:** M. S. Panasiti, A. Mancini, I. Parisi, I. Gualtieri, S. M. Aglioti, F. Mancini

**Affiliations:** ^1^ Department of Psychology Sapienza University of Rome Rome Italy; ^2^ IRCCS Fondazione Santa Lucia Rome Italy; ^3^ Schools of Cognitive Psychotherapy (APC‐SPC) Rome Italy; ^4^ Italian Academy of Schema Therapy Rome Italy; ^5^ Department of Psychology Sapienza University of Rome and CLN^2^S@Sapienza, Istituto Italiano di Tecnologia IIT Rome Italy; ^6^ Guglielmo Marconi University Rome Italy

**Keywords:** altruistic guilt, deception, deontological guilt, disgust, moral behaviour, obsessive–compulsive disorder

## Abstract

Obsessive–compulsive disorder (OCD) is characterised by a dysfunctional sensitivity to a sense of guilt that significantly interferes with everyday functioning and is believed to be a key mechanism in symptom maintenance. Mounting evidence indicates that individuals with OCD are particularly sensitive to deontological guilt, which stems from the perception of violating an internalised rule, as opposed to altruistic guilt, which arises from the feeling of having harmed others. Here, we assess the impact of deontological vs. altruistic guilt on moral behaviour in participants with OCD. Twenty participants with OCD and 20 gender‐ and age‐matched comparison participants took part in a social game in which they could choose to lie for a personal reward (self‐gain lie) or to benefit the other player (other‐gain lie). During the game, they were exposed to stimuli designed to evoke one of three emotional states: deontological guilt (DG), altruistic guilt (AG) or a neutral state. Self‐report ratings of DG and AG evoked by the stimuli were also recorded. Exposure to stimuli that evoke the anticipation of AG was associated with a decrease in self‐gain lies and an increase in altruistic lies in all participants. When individual emotional ratings were taken into account, we found that stronger AG elicited by the stimuli was associated with fewer lies. In contrast, higher DG ratings were associated with a decrease in self‐gain lies in controls but with an increase in self‐gain lies in OCD participants. Our results support the notion that DG is particularly crucial for OCD participants and reveal that it can be particularly disruptive for them, suggesting that this emotion should be a primary target of psychotherapeutic intervention.

## Summary

- **Deontological guilt may have paradoxical effects in OCD.** In individuals with obsessive–compulsive disorder, higher levels of deontological guilt—especially when intertwined with disgust—may paradoxically increase self‐serving and morally questionable behaviours, rather than promoting moral restraint.
- **Not all guilt is clinically equivalent.** Altruistic guilt appears to foster prosocial behaviour across both clinical and non‐clinical populations. In contrast, deontological guilt shows maladaptive effects specifically in OCD, highlighting the importance of distinguishing between different guilt subtypes in assessment and treatment.
- **Clinical interventions should target deontological guilt processes.** Psychotherapeutic approaches for OCD may benefit from explicitly addressing deontological guilt and related self‐disgust processes, as reducing excessive moral self‐condemnation may help prevent maladaptive coping strategies and vicious cycles of guilt‐driven behaviour.

## 1 Introduction

Obsessive–compulsive disorder (OCD) is a debilitating mental condition characterised by intrusive thoughts (i.e., obsessions) and repetitive behaviours (i.e., compulsions) (Diagnostic and Statistical Manual of Mental Disorders, 5th ed., DSM‐5, American Psychiatric Association 2013). Among the various factors contributing to the complexity and severity of OCD, guilt emerges as a significant psychological construct (Shapiro and Stewart 2011). Individuals with OCD commonly experience intense feelings of guilt and responsibility, typically arising from perceived moral violations or anxieties about potentially causing harm to themselves or others (Shafran et al. 1996). Furthermore, OCD participants are generally less tolerant than non‐OCD participants towards the emotion of disgust (for a review, see F. Mancini 2018). Indeed, both fear of guilt and fear of contamination may exacerbate obsessive–compulsive thoughts, perpetuating a vicious cycle of guilt and compulsive rituals (Chiang and Purdon 2019; Olatunji 2010). Research indicates that apprehension regarding guilt correlates with the manifestation of OCD symptoms in non‐clinical samples (Kenny et al. 2023), that disgust propensity is linked to more severe symptomatology in individuals with OCD (Cisler et al. 2010), as well as sensitivity to guilt (Hellberg et al. 2023). Specifically, individuals with OCD appear to be particularly sensitive to deontological guilt (DG), a form of guilt that, according to a dualistic hypothesis, is distinct from altruistic guilt (AG) (F. Mancini and Gangemi 2021). While AG arises from a perceived failure to act in a kind or helpful manner toward others, DG arises from the assumption that one has violated an internalised moral rule, regardless of whether another person is directly affected. This type of guilt is strongly associated with disgust (F. Mancini and Gangemi 2021). Indeed, ‘disgust’ is not just about physical filth. Across the world, people use the same expressions of revulsion to react to social crimes and moral ‘contamination’, such as robbery or incest (Haidt et al. 1997). Importantly, disgust has advanced alongside the emergence of social norms, evolving into a signal that alerts an individual to threats against their personal integrity (Rozin and Fallon 1987). This protective mechanism operates across multiple spheres, influencing not only biological choices like food selection but also complex social interactions and moral conduct (Tybur et al. 2009). The link between DG and disgust has been confirmed both at the subjective and physiological levels. Neuroimaging and behavioural studies reveal that DG is uniquely associated with increased activation in brain regions linked to disgust, such as the insula and anterior cingulate cortex, suggesting overlapping neurobiological substrates for these emotions (Basile et al. 2011). This association plays a particularly prominent role in OCD, where excessive feelings of DG may lead to compulsive cleansing behaviours aimed at alleviating moral contamination (Mancini and Gangemi 2021). Moreover, experimental studies have shown that inducing disgust can enhance deontological moral reasoning, further supporting their bidirectional influence (Ottaviani et al. 2018).

Conversely, AG arises when a person feels they could have acted differently to prevent a negative outcome for another (Castelfranchi 1994). This emotion is deeply rooted in the affective connection and empathy felt toward the victim (Carnì et al. 2013). It represents a state of ‘empathic distress’ where the individual's internal dialogue focuses exclusively on the pain caused to the victim, creating a powerful drive for reparative action (Basile and Mancini 2011). Importantly, it has been shown that those who have a higher propensity to altruistic guilt feelings are also less sensitive towards contamination disgust (A. Mancini, Granziol, Migliorati et al. 2022). This is in line with the notion that altruism and disgust possibly evolved as part of contrasting motivational systems; disgust responding to the need to avoid pathogens and altruistic guilt (via empathy) to the need of providing care to group members or kin (Steinkopf 2017). Furthermore, when examining AG and DG through the lens of moral foundations theory—which identifies six core moral dimensions that individuals use to evaluate right and wrong (Graham et al. 2013)—the two kinds of guilt appear to correspond to violations of distinct moral foundations (A. Mancini, Granziol, Migliorati, et al. 2022). Indeed, people who were more prone to experience AG also exhibited higher scores in the moral pillars of non‐harm (i.e., to avoid causing others suffering) and justice (i.e., treating others impartially) of the Moral Foundation Questionnaire (Glenn et al. 2009). On the other hand, people who were more prone to experience DG also showed higher scores in the pillars of authority (i.e., respect for social order and hierarchies) and purity (i.e., avoidance of physical and spiritual contamination and degradation) (A. Mancini, Granziol, Migliorati, et al. 2022). Previous studies have shown that participants with OCD are particularly sensitive to DG, rather than AG, as they: (i) show different neural activations compared to individuals without OCD in response to stimuli that induce deontological guilt (but not AG stimuli) (i.e., to be looked at by an angry face associated with sentences like ‘How could you behave so immorally!’ during typical target trials) (Basile et al. 2014) and (ii) are more likely than comparison participants without OCD to make deontological choices in the trolley dilemmas (i.e., they are less likely to decide to kill one person to save many others) (F. Mancini and Gangemi 2015). Moreover, the induction of deontological guilt has been found to increase OC‐like symptoms, such as checking and washing behaviours (D'Olimpio and Mancini 2014; Giacomantonio et al. 2019), and to prevent prosocial behaviour, such as altruistic punishment, during a third‐party ultimatum game (A. Mancini and Mancini 2015). This is a variant of the classic Ultimatum Game—where one player proposes how to split money with another, and the second can accept or reject the offer (if they reject, both get nothing)—but with a third party, who is asked to decide on behalf of the second player.

Guilt can regulate moral behaviour in a preventive and anticipated way (influencing the evaluation of the alternatives to a given choice) and in a reparative way (providing feedback and redirecting future actions) (Tangney et al. 2007). In our previous studies, employing an ecologically valid paradigm wherein participants are tempted to deceive another individual for obtaining a personal economic reward (Panasiti et al. 2011), we demonstrated that interacting with a guilt‐inducing partner, because of a low socioeconomic status (Schepisi et al. 2020) or of genuinely good intentions (Azevedo et al. 2018), led to a reduction in self‐serving deception. Additionally, we found that individuals with high manipulative traits, known to lack feelings of guilt for lying, exhibited weaker motor cortical inhibition (Panasiti et al. 2014), reduced activation of the autonomic nervous system (Panasiti et al. 2016), and decreased activation in the anterior cingulate cortex (Dupont et al. 2023) prior to lying for self‐serving purposes. In a similar vein, studies indicate that the anticipation of guilt positively predicts many types of prosocial behaviours (Elgaaied 2012; Lindsey et al. 2007). Interestingly, we recently reported that the induction of deontological guilt enhanced self‐gain deception, particularly among individuals with high disgust sensitivity (Parisi et al. 2021), suggesting that this type of guilt might have detrimental rather than ameliorative effects on moral behaviour. This is particularly relevant for participants with OCD who are highly sensitive to both deontological guilt and disgust (Bhikram et al. 2017; D'Olimpio and Mancini 2014) and may interpret experiences of deontological guilt as evidence of poor moral character—potentially reinforcing a vicious cycle in which feeling immoral leads to behaving immorally. To cast light on this issue, here we tested whether the anticipation of DG, rather than AG, influences the actual moral behaviour of OCD participants more than that of comparison participants without OCD. We anticipated that comparison participants without OCD would decrease their deceptive behaviour following the anticipation of both types of guilt. However, we hypothesised that participants with OCD would exhibit a pattern similar to that shown in our previous study by participants without OCD and with high disgust sensitivity (Parisi et al. 2021), namely an increase in self‐serving deception following DG induction.

**Table:** 

Rationale	Hypothesis	Expected outcome
Guilt inductions often reduce self‐serving behaviour in non‐clinical populations (e.g., Schepisi et al. 2020; Azevedo et al. 2018; Elgaaied 2012; Lindsey et al. 2007)	H 1. Comparison participants without OCD will decrease self‐serving deception after both altruistic guilt (AG) and deontological guilt (DG) inductions	Lower incidence of lying for personal gain in comparison participants without OCD following AG and DG inductions
Previous findings on participants with high disgust sensitivity (Parisi et al. 2021) suggest deontological guilt may paradoxically elevate deceptive behaviour	H 2. Being highly sensitive to disgust, individuals with OCD will increase self‐serving deception following DG induction	Higher incidence of lying for personal gain in OCD participants under DG induction

## 2 Methods

### 2.1 Participants

Based on the effect sizes obtained in previous research where the same experimental paradigm and similar manipulations were used (eta squared = 0.1) (Panasiti et al. 2011), we performed a power analysis (power = 0.80; α = 0.05) for a 2 (between‐subject factor: OCD and comparison gGroup) × 2 outcome (within‐subject factor: favourable and unfavourable) × 3 condition (within‐subject factor: neutral, deontological and altruistic) subjects design with the pwr R function that estimated a sample of two groups of *N* = 18 each to be adequate for testing the three‐way interaction. We collected data from 20 participants per group.

Individuals with OCD (*n* = 20, 10 females; M age, 30.35; SD, 9.86) were recruited from the outpatient clinic of the Cognitive Psychology Association (APC, Viale Castro Pretorio, 116, 00185 Rome RM). Comparison participants without OCD (*n* = 20, 10 females; M age, 30.4; SD, 9.93) were recruited from the general population. We recruited individuals aged 18–65 in both groups. The main inclusion criteria in the experimental group were a primary diagnosis of OCD according to the Diagnostic and Statistical Manual of Mental Disorders, Fifth Edition (DSM‐5; APA, 2013), as determined through a valid clinical evaluation. Exclusion criteria in both groups included major neurological and/or mental disorders and active substance use‐related disorders. Comparison participants without OCD were recruited through our laboratory's internal database and through ongoing study dissemination on social media. OCD and comparison participants did not differ in years of education (t(35.89) = −0.005, *p* = 0.996), occupational status (χ^2^(5) = 3.94, *p* = 0.56), or marital status (χ^2^(4) = 2.82, *p* = 0.59).

Prior to participation, all subjects received an information sheet regarding the study and provided their consent to participate and for the processing of their personal data. They were also informed that their cooperation was voluntary and that they could withdraw from the experiment at any time. After completion of the study, all participants were fully debriefed about the true aims of the experiment.

The experimental protocol was approved by the independent Ethics Committee of Guglielmo Marconi University and conducted in accordance with the principles of the 1964 Declaration of Helsinki. This study was not preregistered, as it included exploratory analyses aimed at refining the study hypotheses.

### 2.2 Experimental Procedure

Participants performed an adapted version of the *Temptation to Lie Card Game* (TLCG; Panasiti et al. 2011; Scattolin et al. 2023; Vabba et al. 2022), a two‐player social paradigm designed to elicit moral conflict by providing the opportunity to deceive another person for monetary gain. In each trial, two cards—an ace of hearts (gain) and an ace of spades (loss)—were initially presented face down. After a fixation cross (500 ms), the cards appeared on the screen and were unveiled after 500 ms, with left/right positions counterbalanced. One card then increased in size after 250 ms, indicating the opponent's (OP) choice. Crucially, only the participant (P), acting as outcome communicator, could see the outcome and was required to communicate it to the OP by selecting either the left or right card via keyboard response (‘A’ or ‘L’), thereby deciding whether to tell the truth or lie. The TLCG is a zero‐sum game: when the OP selected the winning card, the outcome was unfavourable for the participant and lying produced a *self‐gain lie*; when the OP selected the losing card, the outcome was favourable for the participant and lying produced an *other‐gain lie*. Participants were informed that each choice was associated with a variable and unknown monetary amount to prevent trial‐by‐trial gain/loss computations. The task was administered online via Psytoolkit (Stoet 2017), with OP behaviour fully simulated by the computer; participants were told that we recorded the OPs' draws in a separate session and each OP would have been informed about the final outcome of their game. Participants were matched with a different, numerically identified opponent in each block to avoid influences related to personal characteristics. An experimenter supervised the procedure via video call to ensure task comprehension.

**FIGURE 1:**
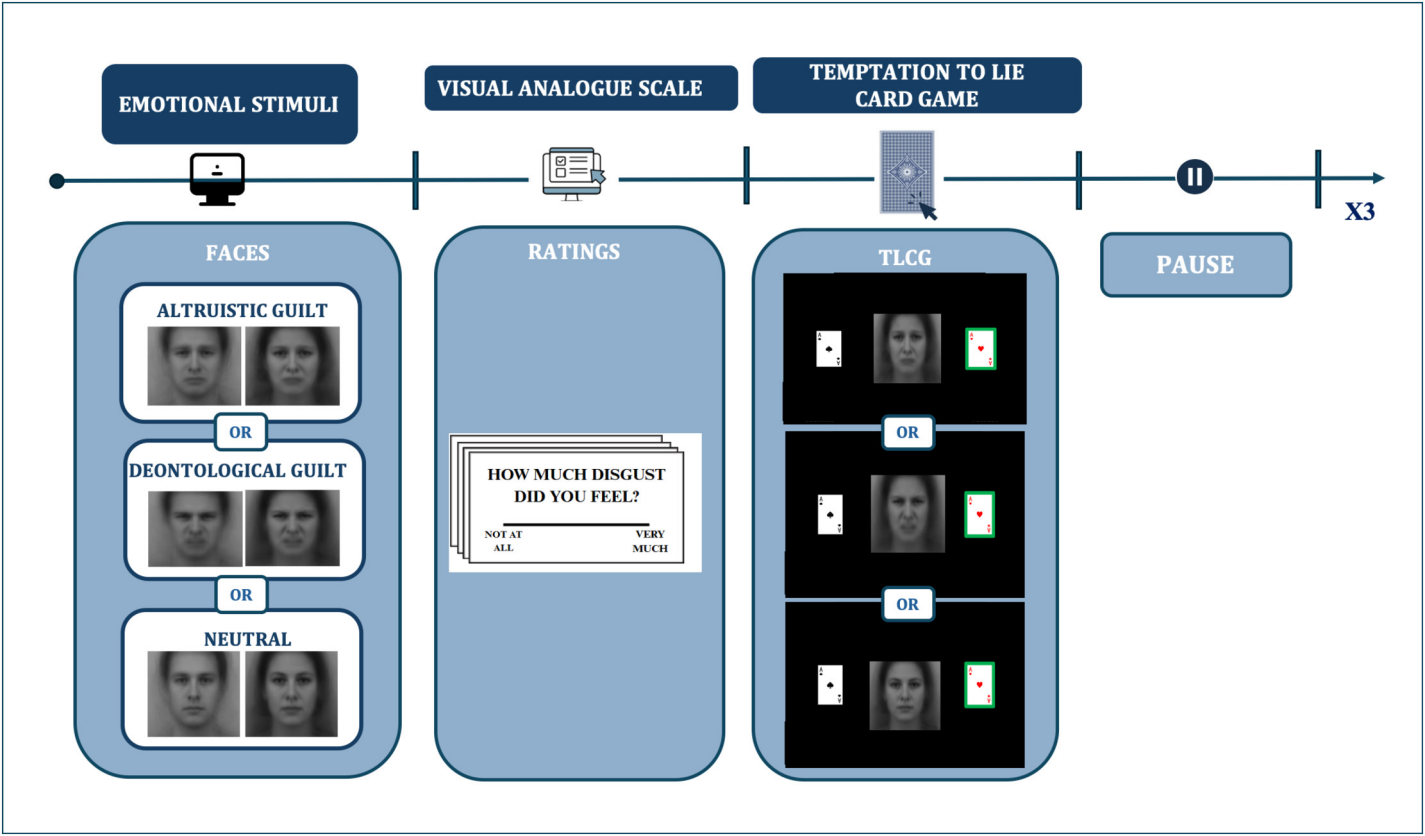
Experimental task structure and trial procedure. The figure illustrates the overall structure of the experimental paradigm. Each block began with the presentation of emotional facial stimuli belonging to one of three conditions: altruistic guilt, deontological guilt or neutral. At the beginning of each block, participants rated how much each face elicited specific emotions using a visual analogue scale (VAS). Participants then completed the Temptation to Lie Card Game (TLCG), during which emotional faces were displayed concurrently with the card game to induce the target emotional context. After a short pause, the procedure was repeated for each condition (three blocks in total).

Each participant completed the TLCG three times (Figure 1), once per emotional condition (neutral, altruistic guilt, deontological guilt), with condition order randomised and counterbalanced. To justify the presence of emotional faces during the game, participants were informed that the aim of the experiment was to investigate how emotional stimuli experienced during social interactions might interfere with performance on a simultaneous task. According to this cover story, participants were told that their primary task was to press the spacebar as quickly as possible whenever the two cards were identical—an event that, unbeknownst to them, occurred four times per block and served solely as an attentional check.

**FIGURE 2:**
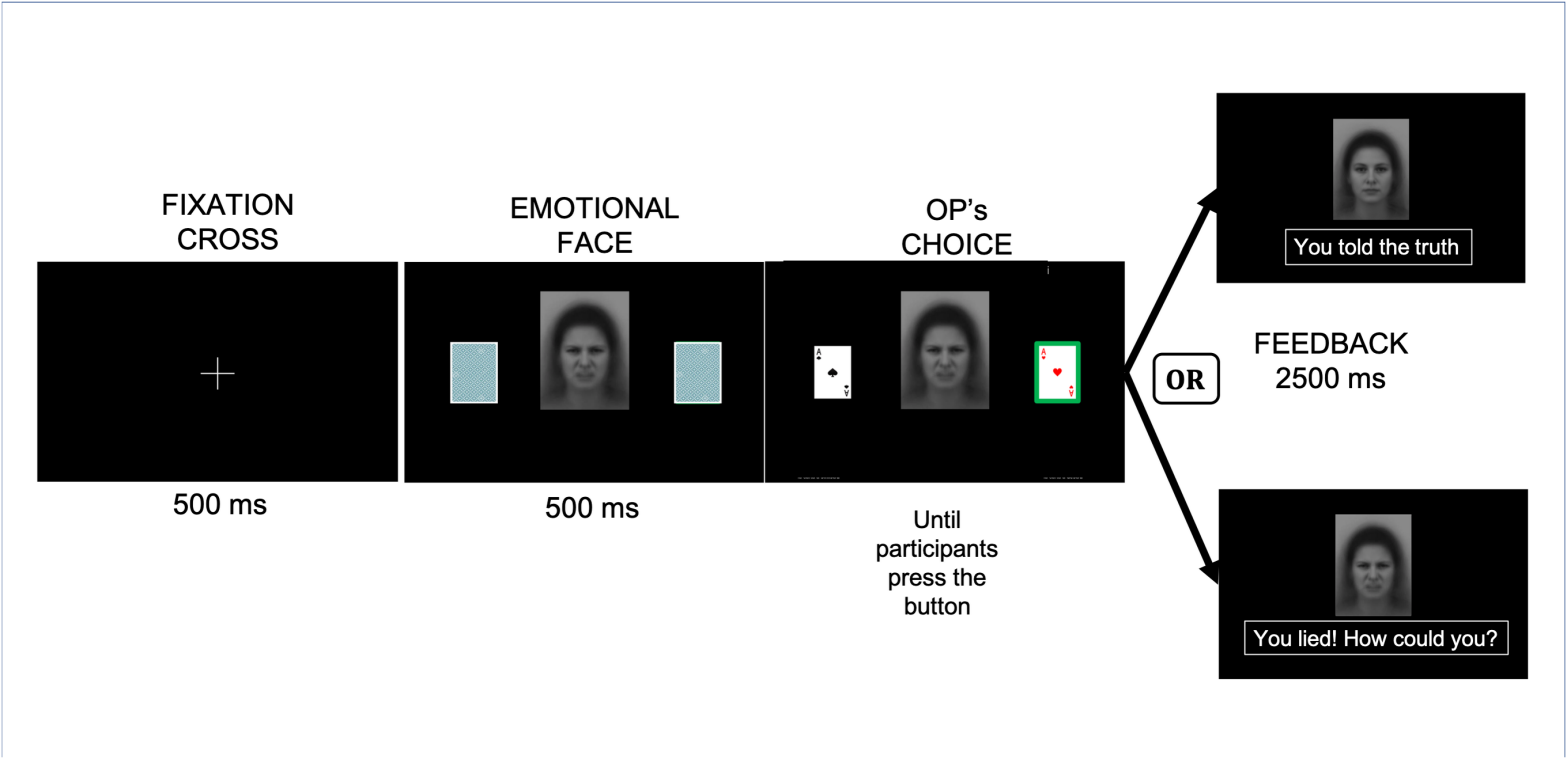
Structure of a single experimental trial. The figure illustrates the temporal sequence of events within a single trial of the Temptation to Lie Card Game (TLCG), shown here for the deontological guilt condition. Each trial began with a fixation cross displayed for 500 ms, followed by the simultaneous presentation of the emotional facial stimulus and two covered cards for 500 ms. One card then indicated the opponent's (OP's) choice, and participants were required to respond by selecting the card to be assigned to the opponent; the emotional face remained visible on the screen until the participant's response. Following the decision, feedback was presented for 2500 ms, consisting of a written message paired with either a neutral or an emotionally congruent facial expression, depending on whether the participant told the truth or lied.

**FIGURE 3:**
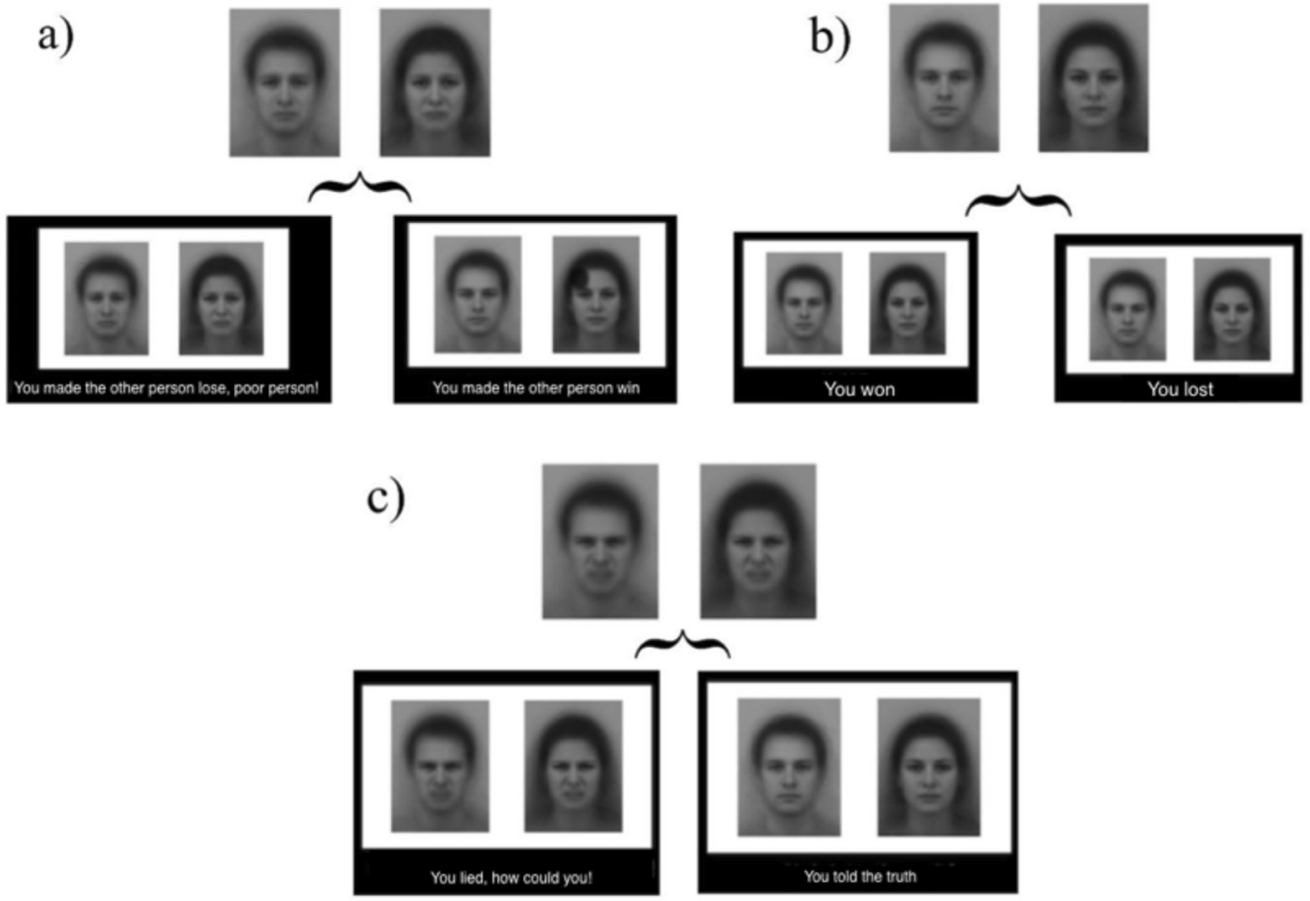
Emotional induction and feedback structure across experimental conditions. The figure illustrates the emotional facial stimuli and feedback messages used in the three experimental conditions. (a) Altruistic guilt condition: sad facial expressions were presented. Following participants' decisions, egoistic choices (self‐gain lie or self‐gain truth) were followed by feedback indicating harm to the other player (‘You made the other person lose, poor person!’) paired with a sad face, whereas altruistic choices (other‐gain lie or other‐gain truth) were followed by feedback indicating benefit to the other player (‘You made the other person win’) paired with a neutral face. (b) Neutral condition: neutral facial expressions were presented throughout the trial, and feedback consisted of outcome‐related messages (‘You won’/‘You lost’) paired with neutral faces. (c) Deontological guilt condition: facial expressions combining anger and disgust were presented. Lying responses were followed by reproachful feedback (‘You lied, how could you!’) paired with an angry/disgusted face, whereas truthful responses were followed by affirming feedback (‘You told the truth’) paired with a neutral face.

At the beginning of each trial, simultaneously with the appearance of the covered cards, a facial expression (male or female) was displayed at the centre of the screen and remained visible until the participant's decision (Figure 2). In the neutral condition, the face showed a neutral expression, and feedback consisted of a written sentence (‘You won’/‘You lost’) paired with the same neutral face. In the altruistic guilt condition, the face initially displayed sadness; following the participant's decision, egoistic choices were followed by ‘You made the other person lose, poor person’ paired with the sad face, whereas altruistic choices were followed by ‘You made the other person win’ paired with a neutral face. In the deontological guilt condition, the initial face displayed a morph of anger and disgust; any lie was followed by ‘You lied, how could you!?’ paired with the angry/disgusted face, whereas truthful responses were followed by ‘You told the truth’ paired with a neutral face. This feedback procedure was designed to selectively reinforce altruistic morality and deontological morality and to link the emotional faces to participants' moral behaviour, mirroring a process of socio‐emotional learning hypothesised to occur during childhood. Specifically, immoral behaviour may have been repeatedly associated with parental feedback characterised either by contempt, moral criticism and reprimand (in the case of deontological morality), or by expressions of sadness and concern for the victim of the transgression (for altruistic morality) (Luppino et al. 2023) (Figure 3).

Participants completed 28 experimental trials per emotional condition (half favourable, half unfavourable). Four additional attention‐check trials per block, in which the two cards were identical, required a speeded spacebar response. Participants believed that we were interested in calculating how much the emotional valence of the face could influence this speed task. Actually, those trials served as an attentional check to exclude participants who failed more than one such trial per block; however, this criterion was never met, and no participants were excluded. Prior to the experimental phase, practice trials were administered until full task comprehension was demonstrated. At the beginning of each emotional block, before task execution, participants rated each facial stimulus using a visual analogue scale (VAS; 1–100) for shame, sadness, fear, disgust, anger, pity, altruistic guilt, deontological guilt, happiness, efficacy and pride.

At the end of each TLCG, participants were asked: ‘Did you feel involved in the game even if you were not sitting in the same room as the other player?’ and ‘To what extent did you feel involved in the game?’ Responses to the first question were provided by selecting ‘Yes’, ‘No’ or ‘I don't know’, while responses to the second question were given on a 5‐point Likert scale (1 = ‘not at all’, 5 = ‘very much’). Participants were excluded if they responded ‘No’ to the first question, reported a score lower than 3 on the second question, or spontaneously expressed skepticism regarding the realism of the game. Applying these criteria, no participants were excluded from the present study.

### 2.3 Selection of Emotional Stimuli

The faces were selected from the Karolinska Directed Emotional Faces (KDEF) (Lundqvist et al. 2015; 1998); the ones used for the deontological induction were created by morphing together the same identity showing anger and disgust (this mix is indeed able to better induce the sense of deontological guilt). For each emotional induction, half of the stimuli depicted female faces and half male faces, ensuring that the effect of sex was balanced across participants and conditions (Figure 3) (Goeleven et al. 2008).

To build our experimental paradigm, we drew on a previous study in which faces expressing anger or sadness were paired with specific statements to elicit either deontological or altruistic guilt (Basile and Mancini 2011). In this study, the AG‐inducing stimuli were specifically designed using sad facial expressions followed by sentences that implied an unfair advantage (e.g., ‘How unfair! I am doing so well, while she/he is so unlucky!’), while the DG‐inducing stimuli were built using an angry face associated with sentences like ‘How could you behave so immorally?!’. The images of human facial expressions we used as stimuli for sadness, anger and disgust, have been validated as among the best for each basic emotion in terms of recognition rates, intensity and arousal (Goeleven et al. 2008). Building on the premise that altruistic guilt arises when an individual compromises another's well‐being through actions or omissions—thus generating empathic pain toward the other (Mancini and Gangemi 2021)—we employ sadness to evoke altruistic guilt. Our choice was further supported by research showing that seeing expressive faces in pain can elicit discomfort in observers, a response akin to experiencing that suffering oneself. These mechanisms primarily involve the insula and the cingulate cortex, brain regions associated with the perception and processing of both emotional and physical pain (Decety and Jackson 2004). Furthermore, the processing of AG‐inducing facial expression stimuli, as compared to that of DG‐inducing facial expression ones, was associated with the activation of the medial prefrontal cortex (Basile et al. 2011), an area that was previously linked to mind‐reading tasks, empathic guilt and compassion feelings (Moll et al. 2005, 2007). Deontological guilt, on the other hand, arises from the violation of internalised moral norms and is supported by the moral pillars of authority and purity (Mancini and Gangemi 2021). Our assumption was the depiction of anger/reproach concerning the transgression of a moral pillar representing authority, and of disgust to anticipate the contravention of the aspiration to a noble and morally elevated life since a strong disgust reaction is associated with what is perceived as degrading (Glenn et al. 2009). Crucially, research shows that people with OCD often exhibit a heightened sensitivity toward certain facial expressions, particularly anger and disgust (Jhung et al. 2010).

### 2.4 Questionnaires

The questionnaires were administered after the experimental session via the PsyToolkit free online survey website (PsyToolkit: A Novel Web‐Based Method for Running Online Questionnaires and Reaction‐Time Experiments—Gijsbert Stoet 2017).

Disgust Scale Revised (DS‐R) (Van Overveld et al. 2011). It is a 25‐item self‐report questionnaire used to measure individual differences in sensitivity to disgust and to examine the relationships among different kinds of disgust. The Italian adaptation was curated and validated by Giampietro et al. (2019). Participants rate their degree of disgust or repugnance if they were to be exposed to each item, using a 5‐point Likert‐type scale with response options ranging from ‘no disgust or repugnance at all’ (or ‘totally disagree’) to ‘extreme disgust or repugnance’ (or ‘totally agree’). It entails a total score and three subscales: core disgust, which represents a sense of offensiveness and the threat of contamination (α = 0.74); animal reminder disgust, which reflects the aversion to stimuli that serve as reminders of the animal origins of humans (α = 0.74); contamination‐based disgust, which refers to disgust reactions based on the perceived threat of transmission of contagion (α = 0.71).

Obsessive‐Compulsive Inventory revised (OCI‐R, Huppert et al. 2007) as a measure of the basic dimensions commonly represented in OCD. The Italian adaptation was curated and validated by Marchetti et al. (2010). The OCI‐R is an 18‐item self‐report measure where participants are required to rate how much some everyday life events bothered or stressed them during the past month, using a five‐point Likert scale. It is composed of 6 factors: washing (α = 0.60), obsessing (α = 0.80), hoarding (α = 0.77), ordering (α = 0.77), checking (α = 0.76) and mental neutralising (α = 0.61), each one describing a set of behaviours typical of OCD patients.

Moral self‐image scale (MSI, Jordan et al. 2015) is a 9‐item scale where participants have to report their evaluations of how caring, compassionate, fair, friendly, generous, hard‐working, helpful, honest and kind they are on a 7‐point Likert‐type scale, ranging from 1 (Much less ________ than the person I want to be) to 7 (Much more ________ than the person I want to be).

The Moral Orientations Guilt Scale (MOGS; A. Mancini, Granziol, Migliorati, et al. 2022) is a 17‐item measure that assesses different types of guilt propensities according to individuals' moral orientation. The analysis of its latent structure pointed at four factors: ‘moral norm violation’ (MNV), which assesses the fear of having outraged an authority and the attempt to prevent guilt by conforming to moral norms; ‘moral dirtiness’ (MODI), measuring the tendency to experience moral disgust toward oneself; ‘empathy’, specifically assessing the tendency to feel guilty for the misfortune of others; and ‘harm’, measuring the propensity to feel and prevent guilt resulting from harming others. The MOGS has shown good construct validity, and the four subscales and the entire MOGS presented good reliability indices (αMNV = 0.82; αHarm = 0.81; αEmpathy = 0.82; αMODI = 0.70; αTotal = 0.87).

State–trait anxiety inventory (STAI, Spielberger 1970) is a psychometric tool widely used in clinical and research settings, which consists of 40 items. The Italian adaptation was curated and validated by Pedrabissi and Santinello (1989). The questionnaire distinctly evaluates the two main dimensions of anxiety: state anxiety and trait anxiety. State anxiety refers to a person's transient emotional state, characterised by feelings of tension, nervousness and worry, and by increased activation of the autonomic nervous system. It refers to how a person feels ‘here and now’, influenced by specific situations or temporary stressors. Trait anxiety, on the other hand, characterises an individual's stable predisposition to perceive situations as threatening, regardless of context. It reflects a consistent tendency toward worry and the perception of situations as stressful or dangerous.

The Beck Depression Inventory II (BDI‐II Beck et al. 1996) is a self‐report questionnaire consisting of 21 items on a 4‐point Likert scale, ranging from 0 to 3, used to assess the severity of depressive symptoms. Each item represents a symptom or attitude associated with depression, such as sadness, guilt, loss of interest, and changes in sleep or appetite. The Italian version of the BDI‐II has proven to have good internal consistency (alpha = 0.80), as well as good convergent and divergent and criterion validity.

### 2.5 Data Analysis

Data processing was performed with SPSS 25 (IBM) and Statistica 7 Software, with the exception of mixed‐effects models that were performed with the statistical software R (R version 3.3.2, Team, R. C. R: A Language and Environment for Statistical Computing, 2018), with the following packages: *car* for analysis of variance and diagnostic functions; *ggplot2* for graphical data visualisation; *lme4* for fitting generalised linear mixed‐effects models (GLMMs); *lsmeans/emmeans* for computing marginal means and post hoc comparisons; effects for visualising model effects. Given the categorical nature of the ‘lie’ variable (1 = lie; 0 = truth), we employed linear mixed‐effect models utilising the ‘lmer’ function and mixed‐effects logistic regression models using the ‘glmer’ function, both of which are components of the ‘lme4’ package (version 1.1‐23). To determine the appropriate random factor structure and prevent over‐parametrisation of our models, we conducted a principal component analysis (PCA). For PCA, we used the ‘rePCA’ function from the ‘RePsychLing’ package (version 0.0.4) while working with the saturated model to select a model in which all random effects contributed to the variance. To assess the significance of fixed effects and interactions, we performed type III Wald chi‐square tests using the ‘Anova’ function from the ‘car’ package (version 3.0‐8). Post hoc comparisons with false discovery rate (FDR) correction were carried out using the ‘emmeans’ package (version 1.8.6).

## 3 Results

### 3.1 Group Differences in State and Psychopathological Measures

In cases where participants did not complete all questionnaire items or provided invalid responses, their data were excluded from the relevant analyses using listwise deletion. This approach ensured the integrity of the dataset but resulted in varying degrees of freedom across certain statistical tests.

Individuals in the OCD group exhibited a lower mean score on ‘Moral Self Image’ compared to the comparison group (4.73 vs. 5.64, respectively; *t* (39) = −2.47, *p* = 0.018), indicating that OC participants are more dissatisfied with their moral image. Additionally, while no significant difference in disgust propensity was observed between the two groups (*t* (35) = 0.10, *p* = 0.919), individuals with OCD demonstrated higher scores on measures related to specific OCD symptoms such as checking (*t* (35) = 2.32, *p* = 0.026) and washing (*t* (35) = 2.46, *p* = 0.019) compared to comparison participants without OCD. Furthermore, individuals with OCD displayed significantly higher levels of trait anxiety compared to comparison participants without OCD (51.95 vs. 35.95, respectively; *t* (39) = 2.84, *p* ≤ 0.01) (see Table 1).

**TABLE 1:** Questionnaires descriptives and comparisons.

	OC group M (SD)	HC group M (SD)	*t*‐value	*p*
**Moral self‐image**	4.73 (1.27)	5.64 (1.04)	−2.47365	0.018
**Disgust scale**	56.15 (17.23)	55.66 (10.89)	0.10296	0.919
**MNV‐MOGS**	18.68 (5.47)	16 (4.18)	1.66768	0.104
**MODI‐MOGS**	7.15 (2.36)	5.61 (2.11)	2.09233	0.044
**EMPATHY‐MOGS**	15.78 (2.76)	15.27 (4.44)	0.42329	0.675
**HARM‐MOGS**	11.94 (1.98)	12.16 (1.91)	−0.34145	0.735
**Hoarding‐OCI**	3.47 (2.93)	2.27 (2.02)	1.43609	0.159
**Checking‐OCI**	4.21 (3.18)	2.05 (2.36)	2.32426	0.026
**Ordering‐OCI**	3.57 (3.90)	3.77 (3.15)	−0.16977	0.866
**Mental neutralising‐OCI**	1.47 (2.29)	0.5 (0.92)	1.67564	0.103
**Washing‐OCI**	2.57 (3.37)	0.55 (0.92)	2.45883	0.019
**Obsessing‐OCI**	5.31 (3.91)	3.33 (3.61)	1.59768	0.119
**BDI**	14.31 (11.61)	9.88 (8.69)	1.30657	0.199
**STAI‐Y TRAIT**	51.95 (16.50)	35.95 (19.41)	2.83561	<0.001

### 3.2 Emotion Induction Manipulation Check

In order to check the effectiveness of the emotional induction, we analysed the VAS scores through a mixed design repeated measures ANOVA with condition (3 levels: neutral, deontological guilt, altruistic guilt) and emotions (11 levels: shame, sadness, fear, disgust, anger, pity, altruistic guilt, deontological guilt, happiness, efficacy and pride) as within‐subject factor and Group (2: OCD, comparison group) as between‐subject factor.

**FIGURE 4:**
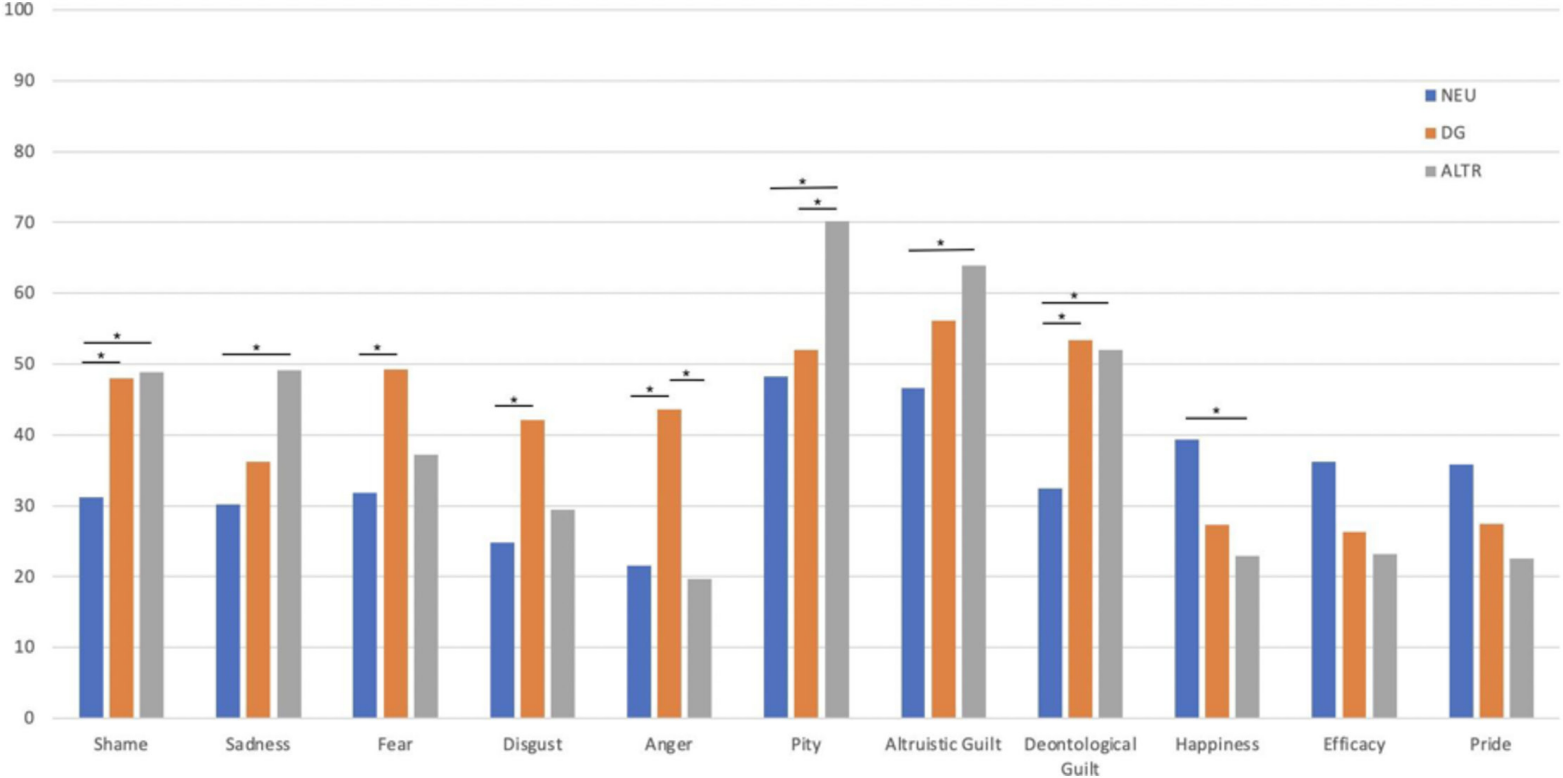
Emotional induction manipulation check: mean scores of self‐reported intensities of each emotion induction for the neutral (blue) deontological guilt (orange) and altruistic guilt (grey) conditions.

A significant condition × emotion interaction emerged, *F* (20,780) = 9.54, *p* < 0.001; η ^2^ = 0.19. Tukey HSD post hoc analyses showed that stimuli used to prime altruistic guilt received a higher score compared to the neutral stimuli, for the following emotions: shame (*p* = 0.01), sadness (*p* = 0.004), pity (*p* < 0.001), altruistic guilt (*p* = 0.01) and deontological guilt (*p* = 0.002). Moreover, altruistic guilt stimuli were rated lower than the neutral stimuli for happiness (*p* = 0.04) and induced higher pity with respect to the stimuli used to induce deontological guilt (*p* = 0.008). The stimuli used to prime deontological guilt were rated higher than the neutral stimuli on the following emotions: shame (*p* = 0.03), fear (*p* = 0.01), disgust (*p* = 0.02), anger (*p* < 0.001) and deontological guilt (*p* < 0.001). Moreover, they induced more anger than the stimuli used to induce altruistic guilt (Figure 4).

### 3.3 Effects of Deontological and Altruistic Guilt on Moral Behaviour

To test H1 and H2 and examine the impact of anticipated guilt on the decision to deceive others for personal or altruistic gain across the two groups, we conducted a multilevel mixed log‐linear regression analysis.

In our model, the dependent variable was the tendency to lie, represented as a dichotomous outcome (lie/truth). The fixed effects included the group (between‐subject factor: OCD and comparison group), outcome (within‐subject factor: favourable and unfavourable), condition (within‐subject factor: neutral, deontological and altruistic) and their respective interactions.

The random effect structure was determined by selecting the most parsimonious random structure by means of PCA (lme4 function *rePCA*) and included the outcome condition as a random slope and the participant as a random intercept.

**FIGURE 5:**
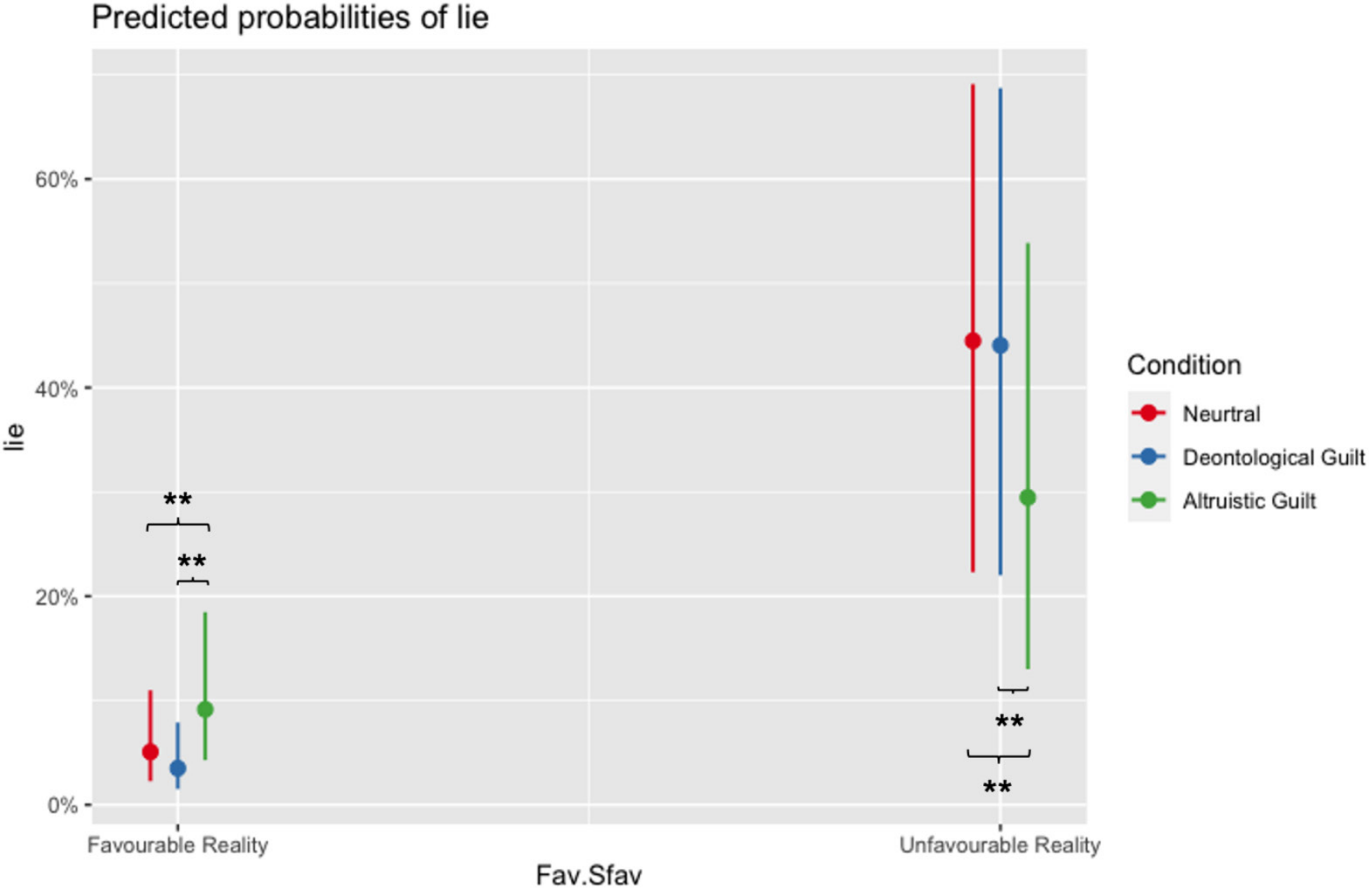
Predicted probabilities of lying. Probability of producing other‐gain (favourable outcome) and self‐gain (unfavourable outcome) lies after the three inductions (neutral, deontological guilt, altruistic guilt). The figure shows the model‐predicted probabilities of lying in favourable and unfavourable outcome contexts (Fav. vs. Sfav), separately for the neutral (red), deontological guilt (blue) and altruistic guilt (green) conditions. Points represent predicted probabilities, and vertical bars indicate 95% confidence intervals. Other‐gain lies were significantly increased by the AG induction, while self‐gain lies were significantly decreased. Asterisks indicate statistically significant pairwise comparisons (**p < 0.01).

The model (*R* ^2^ marginal = 0.129, *R* ^2^ conditional = 0.742) exhibited a significant interaction between outcome and condition (χ^2^ = 14.70, df = 2, *p* < 0.001). This interaction revealed that following the AG induction, participants were less inclined to tell self‐serving lies compared to the neutral condition (estimate = 0.6528, SE = 0.184, *z* ratio = 3.554, *p* = 0.008) and the DG condition (estimate = 0.6316, SE = 0.183, *z* ratio = 3.458, *p* = 0.008). Additionally, participants were more prone to tell altruistic lies after the AG induction as opposed to the neutral condition (estimate = −0.6323, SE = 0.223, *z* ratio = −2.838, *p* = 0.006) and the DG condition (estimate = −1.0214, SE = 0.242, *z* ratio = −4.222, *p* = 0.001) (see Figure 5).

**Table:** 

Effect	χ^2^	*df*	*p*	Significance
Intercept	26.80	1	2.25 × 10^−7^	***
Outcome	7.69	1	0.0056	**
Condition	8.92	2	0.0116	*
Group	0.36	1	0.5482	n.s.
Outcome × condition	14.71	2	0.0006	***
Outcome × group	0.43	1	0.5099	n.s.
Condition × group	0.14	2	0.9302	n.s.
Outcome × condition × group	0.03	2	0.9835	n.s.

### 3.4 Moderation of Perceived Deontological and Altruistic Guilt on Moral Behaviour

Since deontological guilt stimuli and altruistic guilt stimuli increased the ratings of both perceived altruistic and deontological guilt among our participants, we decided to incorporate participants' VAS scores (of perceived deontological and altruistic guilt) as predictors in our model and provide further evidence for both H1 and H2.

In this model, the dependent variable was the tendency to lie, represented as a dichotomous outcome (lie/truth). The fixed effects included the group (between‐subject factor: OCD and comparison group), outcome (within‐subject factor: favourable and unfavourable), perceived deontological guilt (within continuous variable) and its respective interactions with group and outcome, perceived altruistic guilt (within continuous variable) and its respective interactions with group and outcome.

The random effects structure was determined by selecting the most parsimonious random structure by means of PCA (lme4 function *rePCA*) and included the subject as a random intercept.

**FIGURE 6:**
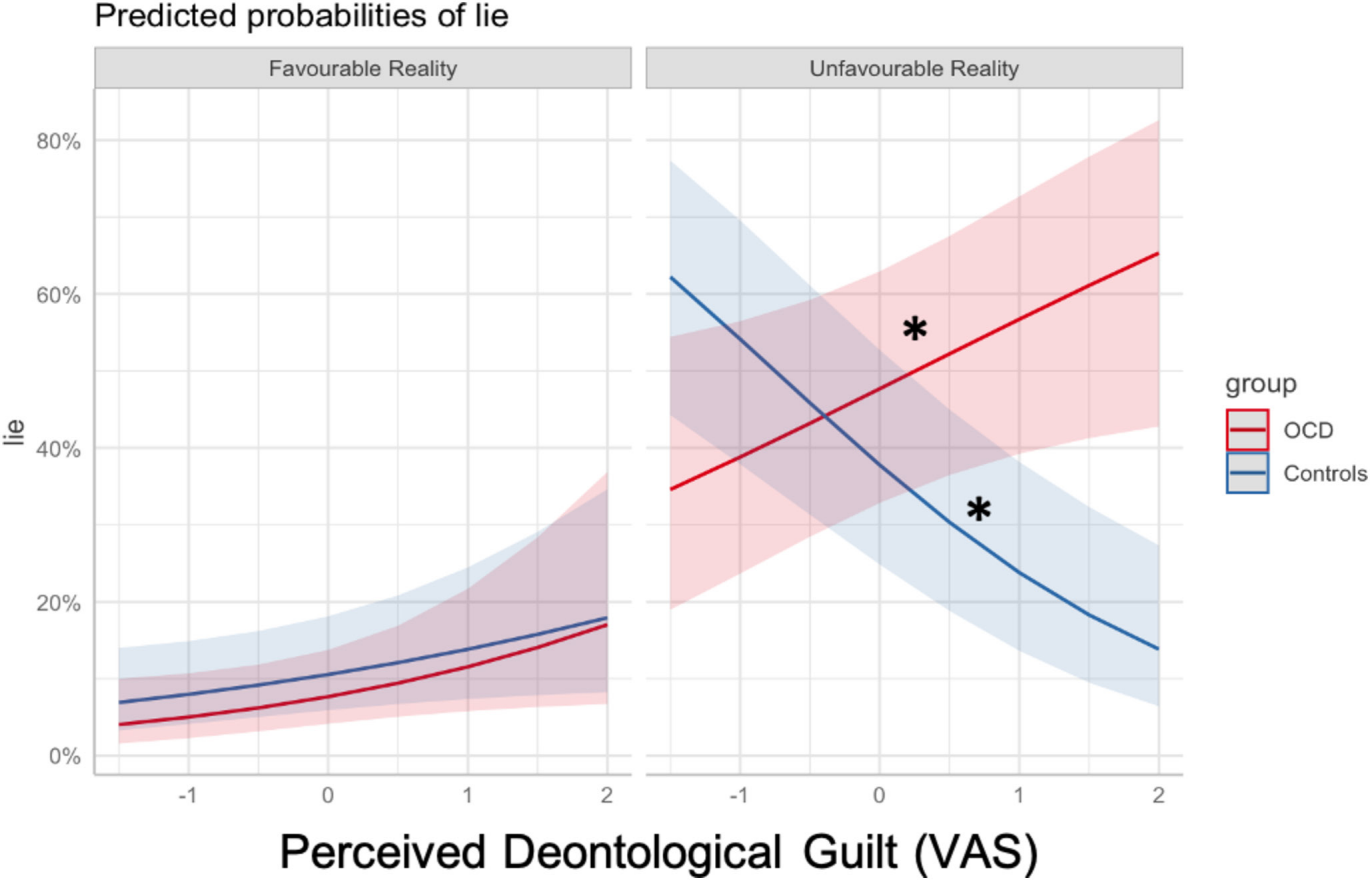
Predicted probabilities of lying as a function of perceived deontological guilt, outcome valence and group. The figure depicts model‐predicted probabilities of lying as a function of perceived deontological guilt (standardised VAS scores), separately for favourable (other‐gain lies) and unfavourable (self‐gain lies) outcome contexts. Predictions are shown for individuals with obsessive–compulsive disorder (OCD; red) and comparison participants without OCD (blue). Shaded areas represent 95% confidence intervals. In unfavourable outcomes, increasing levels of perceived deontological guilt were associated with opposite behavioural patterns across groups, with higher lying probabilities in the OCD group and lower lying probabilities in comparison participants. Asterisks indicate statistically significant group differences (*p < 0.05).

The model (*R* ^2^ marginal = 0.194, *R* ^2^ conditional = 0.482) revealed a significant main effect of perceived altruistic guilt (*χ* ^2^ = 9.52, df = 2, *p* = 0.002) indicating that the higher the perceived AG, as indicated by the score on the corresponding VAS, the lower the number of lies (estimate = −1.0973, SE = 0.3135, *z* value = −3.500, *p* < 0.001). In addition, we found an interaction involving outcome, group and perceived deontological guilt (*χ* ^2^ = 7.52 Df = 1, *p* = 0.006). This interaction unveiled that while in the comparison group higher perceived DG was associated with a decrease in self‐gain deception (estimate = −0.665, SE = 0.146, *z* ratio = −4.567, *p* < 0.001), in the OCD group it was associated with an increase in self‐gain deception (estimate = −0.665, SE = 0.146, *z* ratio = −4.567, *p* < 0.001) (see Figure 6).

**Table:** 

Effect	χ^2^	*df*	*p*	Significance
Intercept	17.48	1	2.91 × 10^−5^	***
Outcome	43.53	1	4.19 × 10^−11^	***
Group	0.00	1	0.9942	n.s.
Perceived DG	4.05	1	0.0441	*
Perceived AG	9.52	1	0.0020	**
Outcome × group	1.08	1	0.2992	n.s.
Outcome × perceived DG	0.12	1	0.7245	n.s.
Fav. vs. Sfav × perceived AG	0.76	1	0.3830	n.s.
Group × perceived DG	0.27	1	0.6008	n.s.
Group × perceived AG	1.14	1	0.2860	n.s.
Outcome × group × perceived DG	7.52	1	0.0061	**
Outcome × group × perceived AG	2.73	1	0.0984	.

## 4 Discussion

Building on our previous report that highlighted the enhancement of self‐gain deception following the induction of deontological guilt in non‐selected participants with high disgust sensitivity (Parisi et al. 2021), we examined the impact of deontological and altruistic guilt on moral behaviour in individuals with OCD and comparison participants without OCD. To this end, we induced different emotional states by exposing participants to visual stimuli (facial expressions) and verbal feedback designed to elicit neutral, DG and AG emotional states, as described in (Basile et al. 2011) while participants performed the TLCG, a social moral decision paradigm in which they could lie to another person in one's own or in the other interest.

### 4.1 Effects of Altruistic and Deontological Guilt on Moral Behaviour

Hypothesis 1 *Comparison participants without OCD will decrease self‐serving deception after both altruistic guilt (AG) and deontological guilt (DG) inductions*.

Partially in line with our hypothesis, we found that AG induction decreased self‐serving lies and increased other‐serving lies. This result is different from what was found in non‐selected participants (Parisi et al. 2021), where the DG induction increased self‐serving behaviour, while AG induction did not modulate moral behaviour. This seeming discrepancy may be accounted for by the fact that the guilt induction was delivered in a different way. Specifically, in the study of Parisi et al. (2021), participants listened to a single audio story designed to induce altruistic guilt, deontological guilt or a neutral emotional state; each story was recorded in the second person (e.g., ‘You are …’) to facilitate immersion in the described scenario and was adapted to the participant's gender. During the listening, participants were simply required to imagine themselves while performing an immoral act, in order to induce a sense of altruistic (e.g., not helping someone in trouble) or deontological (e.g., cheating on someone without telling them) guilt. In contrast, in the present study, participants *anticipated* the guilt they would experience if they lied. Guilt *experience* emerges from the awareness that our previous behaviour did not meet our moral standards (Tracy & Robins, 2004). Specifically, the experience of altruistic guilt is associated with the acknowledgement of having failed one's one altruistic goal (F. Mancini and Gangemi 2021) an experience that is mostly often associated with reparatory behaviours for the victims (Rebega et al. 2013) but also with actions that can be detrimental for other people in the social environment (De Hooge et al. 2011).

Conversely, the *experience* of deontological guilt, which arises from the awareness of having violated an internalised moral principle, appears to induce a tendency toward more selfish rather than altruistic behaviour (Parisi et al. 2021). This phenomenon may be attributed to the nature of deontological guilt, that is closely linked to a negative self‐evaluation intertwined with self‐disgust (Ottaviani et al. 2019), rather than a negative assessment of one's own actions. Consequently, attempting to enhance moral conduct solely through reparative behaviours might not be sufficient to alleviate one's deontological guilt.

In contrast to the *experience* of guilt, the *anticipation* of guilt consists in imagining the consequences of performing a future immoral behaviour. In this study, by priming participants with faces showing specific emotional expressions, we made participants anticipate the consequences of deontological guilt, i.e., being the target of others' disgust or e blame/reproach (Basile et al. 2018; Stevenson et al. 2010) or that of altruistic guilt, i.e., facing the sufferance of the other (F. Mancini and Gangemi 2021). Action tendencies associated with *anticipating* guilt are different from those associated with *experiencing* guilt. Indeed, anticipating the negative consequence of an action is associated with the prevention of that action. Thus, we expected that anticipating deontological guilt could potentially reduce any immoral action (both egoistic and altruistic lies) while anticipating altruistic guilt could potentially curb any egoistic behaviour (both egoistic lies and egoistic truth). Furthermore, we expected the effect of deontological guilt to be opposite for OCD participants. Partially aligning with our expectations, we observed that anticipating altruistic guilt inhibited selfish acts by reducing egoistic lies and increasing altruistic lies (i.e., decreasing egoistic truths). This finding is consistent with previous studies indicating that the anticipation of AG increases many altruistic behaviours such as recycling or donation intentions (Elgaaied 2012; Ming et al. 2019) or helping and altruistic prevention behaviours (Erlandsson et al. 2016; Turner et al. 2023). On the other hand, the anticipation of DG did not elicit a change in participants' behaviour compared to the neutral induction. This result might be due to the fact that lying during the TLCG is somehow allowed by the game context and thus, lying in our task might not be associated with the violation of a deontological norm (such as authority) but rather as a violation of an altruistic norm only (despite its gamified nature, selfish actions still lead to monetary loss for the other participant). On the other hand, it is also possible that the effect of guilt is counteracted by that of disgust, which, as shown by our results on the VAS scores, is also triggered by the deontological guilt induction. Disgust may prompt avoidance and self‐protective behaviours (Curtis et al. 2004) and few studies have shown that it can enhance unethical self‐serving decisions (Kugler et al. 2021; Lim et al. 2015; but also see Winterich et al. 2014 for a lack of replication), ultimately resulting in moral decisions that are indistinguishable from those observed during the neutral induction.

### 4.2 Differences Between OCD and the Comparison Group Without OCD on How Perceived Guilt Modulates Moral Behaviour

Hypothesis 2 *Being highly sensitive to disgust, individuals with OCD will increase self‐serving deception following DG induction*.

Crucially, unlike the induction method employed in Parisi et al. (2021), our experimental induction failed to selectively modulate perceived deontological and altruistic guilt. Thus, in a subsequent analysis, we examined the influence of perceived altruistic and deontological guilt on moral behaviour. Importantly, because these analyses are based on self‐reported perceived guilt rather than the experimental manipulation per se, they should be interpreted with caution, as they are correlational and potentially sensitive to unmeasured third variables.

These results aligned perfectly with our hypothesis, revealing that perceived altruistic guilt was linked to a universal decrease in dishonesty across both groups, while perceived deontological guilt had divergent effects on the moral behaviour of OCD participants and comparison participants without OCD. Specifically, whereas in participants without OCD, higher perceived deontological guilt was associated with fewer self‐gain lies, in OCD participants, it was related to a greater frequency of self‐gain lies. This effect was comparable to the positive association between disgust sensitivity and self‐gain lies that we found in a previous study, after the deontological induction (Parisi et al. 2021). We believe this can be explained by the affect‐as‐information bias, a psychological mechanism whereby individuals use their emotions as critical information to form judgements about the world or themselves, rather than referencing objective reality (Gangemi et al. 2021). In our case, participants with OCD might interpret their perceived sense of deontological guilt as indicative of their own morality (‘Since I feel immoral, I am immoral’) and consequently behave dishonestly. This mechanism is prevalent in various psychopathological conditions (Paredes‐Mealla et al. 2023), in people with high sensitivity to guilt (Gangemi et al. 2007), and in people with high sensitivity to disgust (Verwoerd et al. 2013). Consistent with the hypothesis that deontological guilt, like disgust, shifts attention toward one's own internal moral standing (the Self), recent results showed that AG and DG have different modulating effects on empathy tasks at both the behavioural and neural levels (Zhang and Chen 2026). Specifically, at the behavioural level, participants in the DG condition reported lower empathy scores and slower reaction times when identifying others' pain compared to those in the AG condition. At the neural level, using event‐related potentials (ERPs), the study observed that DG reduced early neural responses to others' pain (specifically the N110 and P2 components). In contrast, AG showed a stronger association with late‐stage cognitive processing of empathy (P3 or LPP), suggesting that altruistic guilt keeps the individual cognitively engaged with the other person's feelings.

Furthermore, studies suggest that the association between DG and OCD traits is better understood when considering moral instability. Specifically, obsessive traits are linked to greater instability in one's moral sense of self and increased vulnerability in moral status (Giacomantonio et al. 2024). When an OCD participant simultaneously experiences fear of guilt and fear of being immoral, they will employ every possible strategy to maintain a stable high moral status and prevent a ‘moral fall’, even if it involves costly compulsions such as repeated checking or washing. Accordingly, urge to act and obsessive doubts levels are positively associated with ‘feared‐self’ levels (Yang et al. 2021), which in OCD participants corresponds to the fear of being impure, immoral, and bad (Aardema et al. 2021). In our experiment, however, OCD participants lied more in an egoistic way as a function of how much they felt already deontologically guilty and putatively already perceived their moral status as low. Participants without OCD, who are probably less scared to ‘fall’ on a moral hierarchy, are probably simply guided by a more altruistic goal, even with a lower moral status. Interestingly, a very similar result has been shown by a study conducted in participants with obsessive–compulsive personality disorder (OCPD) where, during economics games, participants showed a weaker guilt‐induced cooperation and less guilt‐induced compensations to the victims (Xiao et al. 2023), suggesting that clinical conditions characterised by a pathological sense of guilt might show paradoxical effects of this emotion on the subsequent interpersonal behaviour. This aligns with observations that a positive association between moral norm violation, guilt, and obsessive–compulsive (OC) traits is present only in OCD participants and is non‐significant in non‐OCD participants (e.g., anxious or depressed individuals). Furthermore, in OCD participants, the propensity to feel degraded and dirty while feeling guilty (i.e., moral dirtiness guilt) was positively associated with depression levels. Interestingly, for non‐OCD participants, depression was more closely related to altruistic guilt propensity (A. Mancini, Granziol, Gragnani, et al. 2022). These data support the perspective that a loss of moral status may be particularly devastating for OCD participants, paradoxically prompting actions focused on the self rather than orienting it proactively towards the other.

### 4.3 Clinical Implications

Taken together, this evidence suggests that in OCD participants, an exaggerated sense of guilt, especially when mixed with disgust as in deontological guilt, can prompt maladaptive coping strategies. These strategies may instigate vicious cycles beginning with the feeling of being immoral, which might be interpreted as actual immorality, and subsequently trigger immoral behaviour. This decline in moral behaviour may, in turn, reinforce the initial perceived sense of immorality, leaving these participants burdened with a sense of guilt that is not easily reversible. This interpretation is also in line with the results shown by the questionnaire in our study, which show that OCD participants present significantly lower moral image and a higher tendency to feel deontological guilt compared to the comparison group without OCD.

## 5 Conclusions and Limitations of the Study

Overall, our findings shed light on the significant impact of both altruistic and deontological guilt on moral behaviour, demonstrating that the anticipation of both kinds of guilt can be associated with different tendencies in moral behaviour.

Importantly, our results suggest relevant clinical implications for individuals with obsessive–compulsive disorder, indicating that the induction of deontological guilt may be particularly counterproductive in this population. At the same time, these conclusions must be interpreted with caution. Several methodological limitations should be acknowledged, including the relatively small sample size, the heterogeneity of OCD symptoms treated as a single group, reliance on self‐report measures, and the online format of the task, which may not fully capture the complexity of real‐world moral dilemmas. Moreover, some key effects emerged from correlational analyses based on participants' perceived guilt rather than from the experimental manipulation. As such, these findings do not allow for strong causal inferences and may be sensitive to unmeasured third variables.

Despite these limitations, the present results are consistent with the notion that deontological guilt may play a role in the development and maintenance of OCD psychopathology (Basile et al. 2018). This highlights the importance of targeting deontological guilt‐related processes in psychotherapeutic interventions, while underscoring the need for future studies employing stronger experimental manipulations and designs better suited to causal inference.

## Funding

MSP was supported by the Young Researchers Grant, Italian Ministry of Health (2021‐12372815); SMA was supported by ERC Advanced Grant eHONESTY (789058).

## Data Availability

Data will be made available upon request.
